# Local and network-level dysregulation of error processing is associated with binge drinking

**DOI:** 10.1016/j.nicl.2021.102879

**Published:** 2021-11-09

**Authors:** Austin B. Alderson Myers, Donatello Arienzo, Sean M. Molnar, Ksenija Marinkovic

**Affiliations:** a Department of Psychology, San Diego State University, 5500 Campanile Dr., San Diego, CA 92182, USA; b Department of Radiology, University of California, San Diego, 9500 Gilman Dr., La Jolla, CA 92093, USA

**Keywords:** Error processing, Cognitive control, Binge drinking, Go/NoGo, Functional magnetic resonance imaging, Functional connectivity

## Abstract

Binge drinking refers to the pattern of alcohol consumption that brings blood alcohol levels to or above legal intoxication levels. Commonly practiced by young adults, it is associated with neurofunctional alterations, raising health-related concerns. Executive deficits may contribute to the inability to refrain from excessive alcohol intake. As a facet of cognitive control, error processing allows for flexible modification of behavior to optimize future outcomes. It is highly relevant to addiction research, as a failure to inhibit excessive drinking results in relapses, which is a hallmark of alcohol use disorder. However, research on local and system-level neural underpinnings of inhibition failures as a function of binge drinking is limited. To address these gaps, functional magnetic resonance imaging (fMRI) was used to examine local changes and interregional functional connectivity during response inhibition errors on a Go/NoGo task. Young adult binge drinkers (BDs) performed equally well as light drinkers (LDs), a group of demographically matched individuals who drink regularly but in low-risk patterns. In contrast, BDs exhibited greater fMRI activity to inhibition errors contrasted with correct NoGo trials in the rostral anterior (rACC) and posterior cingulate cortices (PCC), as well as right middle frontal gyrus (R-MFG). Furthermore, BDs showed increased connectivity between the rACC and right lateral prefrontal cortex, in addition to greater connectivity between the R-MFG and the left ventrolateral and superior frontal cortices. Imaging indices were positively correlated only with alcohol-related measures, but not with those related to moods, disposition, or cognitive capacity. Taken together, greater error-related activity and expanded functional connectivity among prefrontal regions may serve a compensatory role to maintain efficiency of inhibitory control. Aligned with prominent models of addiction, these findings accentuate the importance of top-down control in maintaining low-risk drinking levels. They provide insight into potentially early signs of deteriorating cognitive control functions in BDs and may help guide intervention strategies aimed at preventing excessive drinking habits.

## Highlights

• Go/NoGo performance does not differ between binge (BDs) and light drinkers.
• BDs show greater BOLD activity to inhibition errors primarily in prefrontal areas.
• Greater functional connectivity in the frontal cortex correlates with drinking.
• Observed increase in error-related activity may serve a compensatory role.
• This is consistent with allostatic hyperexcitability reflecting neuroadaptation.

## 1 Introduction

Binge drinking, also termed heavy episodic drinking, refers to engaging in episodes of excessive alcohol intake followed by periods of withdrawal. A binge episode is typically defined as consuming 4+/5+ (women/men) standard alcoholic beverages within a two-hour window, which commonly elevates BAC to the legal limit of 0.08 g/dL or higher (Courtney and Polich, 2009, National Institute on Alcohol Abuse and Alcoholism, 2017, Patrick and Terry-McElrath, 2017). However, alcohol intake in young adults frequently surpasses this definition benchmark, resulting in much higher BAC levels, which exacerbates alcohol’s neurotoxic effects (Naimi et al., 2010, Patrick and Terry-McElrath, 2017, Petit et al., 2014b). Binge drinking is associated with various health risks and an increased likelihood of engaging in impulsive and potentially unsafe behavior (Carbia et al., 2017, Crews et al., 2016, Hingson et al., 2017, Koob and Volkow, 2016, Townshend et al., 2014). Binge drinking pattern is prevalent among young adults, peaking during early 20-ies, and typically declining as individuals age and assume roles associated with later adulthood (Patrick et al., 2019). Nonetheless, some binge drinkers (BDs) continue consuming alcohol at elevated levels (Witkiewitz et al., 2014). Longitudinal studies demonstrate that binge drinking during college is a significant predictor of alcohol use disorder (AUD) development 10 years later (Jennison, 2004, O'Neill et al., 2001). Some models conceptualize binge drinking as a transitional stage in a cyclic process leading to compulsive intake (Kimbrough et al., 2017, Koob, 2013, Koob and Le Moal, 2008). However, the shift from impulsive to compulsive consumption is multiply determined and understanding of the neural underpinnings of this process is limited.

Prominent accounts include executive dysregulation as an especially relevant dimension of the dynamics of addiction disorders (Baler and Volkow, 2006, Koob, 2011, Le Berre et al., 2017). Primarily subserved by the frontal lobes, executive functions include an array of capacities that make it possible to efficiently choose behaviors that promote achieving goals and intentions while respecting situational constraints (Alvarez and Emory, 2006, Carter et al., 2000, Diamond, 2013). Repeated cycles of high-intensity drinking exert deleterious effects on the prefrontal networks, which may further reduce the ability to inhibit seeking and consuming alcohol at hazardous levels (Crews and Boettiger, 2009, Goldstein and Volkow, 2011, Koob, 2011, Koob and Volkow, 2016, Loeber et al., 2009a, Loeber et al., 2009b, Oscar-Berman and Marinković, 2007, Parvaz et al., 2011). Individuals engaging in binge drinking are frequently young, healthy adults who very rarely seek treatment (Knight et al., 2002). Typically, they believe that binge drinking is socially acceptable at that age (Sudhinaraset et al., 2016), rendering them unaware that their drinking may be problematic, or that it even impacts their cognitive functioning or self-control (Bishop and Rodriquez Orjuela, 2018, Probst et al., 2015). And yet, inhibition failures on tasks probing inhibitory control are predictive of an escalation in binge drinking, AUD severity (Clark et al., 2017, Claus et al., 2013, Nigg et al., 2006, Paz et al., 2016, Paz et al., 2018), as well as relapse in cocaine dependence (Luo et al., 2013).

Two major inhibitory tasks (Go/NoGo and Stop-Signal) are commonly used to probe the ability to inhibit an initiated motor response. During the Go/NoGo response prepotency is established by frequent, rapidly presented Go trials (Wessel, 2018b), whereas during the Stop-Signal task each trial is a Go up to the point a Stop-Signal (e.g., a tone) is potentially presented (Logan et al., 1997). The requirement to withhold the already initiated Go responses on unpredictable, infrequent NoGo or Stop trials often elicits inhibition failures, making both tasks well suited for studying error monitoring (Wessel, 2018a). The ability to process errors is considered a fundamental aspect of cognitive control that allows for flexible modification of behavior to optimize future decisions (Krönke et al., 2018, Neta et al., 2015, Wessel, 2018a). It is highly relevant to addiction research, as a failure to inhibit excessive drinking results in relapses, which is a hallmark of alcohol use disorder (Koob and Volkow, 2016, Luijten et al., 2014, Marhe et al., 2014, Marhe et al., 2013). The dearth of neuroimaging studies investigating the neural underpinnings of error monitoring in alcohol misuse is surprising. The extant evidence indicates that individuals with AUD show greater fMRI blood oxygen level-dependent (BOLD) activation to inhibition failures in bilateral frontal cortices relative to control participants (Li et al., 2009). However, another study has reported a negative association with AUD severity such that individuals with more severe AUD exhibit comparatively reduced prefrontal BOLD response (Claus et al., 2013). In BDs, error-related research is even more scarce, with a single study that reported regionally variable group differences in BOLD activity to errors on a Go/NoGo task superimposed on alcohol-related vs. contextually neutral pictorial stimuli (Campanella et al., 2017) The need for more neuroimaging evidence is further underlined by the fact that behavioral measures are often inadequate in detecting cognitive deficits in BDs on cognitive tasks more broadly. Many studies employ college students who are high-functioning, who perform well on standardized neuropsychological tests, and show subtle or no differences from light drinkers (LDs) on such measures. In contrast, studies using neural measures report robust group differences, emphasizing the benefits of such an approach (Affan et al., 2018, Ahmadi et al., 2013, Ames et al., 2014, Antunes et al., 2020, Campanella et al., 2017, Correas et al., 2019, Crego et al., 2012, Holcomb et al., 2019, Huang et al., 2018, Lannoy et al., 2019, López-Caneda et al., 2017, Maurage et al., 2009, Petit et al., 2014a, Schweinsburg et al., 2010).

Error processing is considered to be a facet of cognitive control that relies on continuous monitoring of actions and their outcomes, resulting in behavioral tuning to minimize failures (Carter and van Veen, 2007, Krönke et al., 2018, Neta et al., 2015, Wessel, 2018a). As a hub of cognitive control processes, the ACC has extensive anatomical connections with the lateral PFC and other areas, supporting its role as a primary region involved in regulating behavior (Barbas, 2000, Devinsky et al., 1995, Paus, 2001, Zhang et al., 2019). Robust engagement of the dorsal ACC (dACC) during errors (Carter and van Veen, 2007, Marinkovic et al., 2012, Marinkovic et al., 2013) is accompanied with the lateral PFC activity to interactively effectuate cognitive control (Botvinick et al., 2004, Kerns et al., 2004), as demonstrated with functional connectivity methods (Botvinick et al., 2004, Danielmeier and Ullsperger, 2011, Dosenbach et al., 2007, Kerns et al., 2004, Marinkovic et al., 2019, Ridderinkhof et al., 2004b, Smith et al., 2019). The rostral ACC (rACC) is additionally activated by errors which may reflect engagement of limbic networks during error-related processing of the motivational or affective salience aspects (Hiser and Koenigs, 2018, Tang et al., 2019). Because the brain functions as an integrated system, (Bullmore and Sporns, 2009), additional insight into network-level changes associated with excessive drinking can be obtained with an event-related functional connectivity (ERFC) approach during error processing. Evidence obtained during cognitive tasks in AUD samples indicates that network dysregulation extends beyond the local activation differences afforded by traditional fMRI analysis (Chanraud et al., 2011, Courtney et al., 2013, Müller-Oehring et al., 2013, Park et al., 2010, Schulte et al., 2012, Seo et al., 2016). This includes reduced fronto-striatal (Courtney et al., 2013, Park et al., 2010) or fronto-midbrain connectivity in the AUD (Müller-Oehring et al., 2013, Schulte et al., 2012) compared to control groups in a variety of tasks. Conversely, greater posterior cingulate-cerebellar (Chanraud et al., 2011) and midbrain-orbitofrontal connectivity (Schulte et al., 2012) has been observed in individuals with AUD. Animal research extends these findings as widespread remodeling of functional connectivity has been observed in alcohol-dependent mice at a cellular level (Kimbrough et al., 2020). Aside from studies reporting dysregulated executive and reward-salience associated networks during rest (Arienzo et al., 2020, Herman et al., 2019, Sousa et al., 2019), it appears that there are no studies on event-related functional connectivity in BDs. A growing body of evidence linking binge drinking to a range of negative outcomes underscores the need for further research on regional neural changes as well as those at a level of an interactive system in BDs. To address these gaps, the overall aim of this study was to employ fMRI BOLD during a Go/NoGo task to a) examine error-specific regional activation patterns, and b) to use ERFC to investigate network-level dysregulation as a function of binge drinking habits.

## 2 Materials and methods

### 2.1 Research participants

Thirty-seven right-handed young, healthy adults (20 female, age 24.47 ± 3.67) were recruited from San Diego State University and the surrounding community. Participants had no history of seizures, traumatic brain injury, neuropsychiatric disorders, hearing, or vision problems. They reported no tobacco, illicit drug, or prescription drug use at least one month prior to scanning, and no previous or ongoing enrollment in alcohol abuse treatment programs. BD and LD group assignment was based on a screening questionnaire assessing rate, frequency, quantity, and pattern of alcohol consumption. Binge episodes were defined as occasions of consuming 6+/5 + drinks within a 2-hour time span for males and females respectively (Lange and Voas, 2001). This criterion has been shown to produce a blood-alcohol concentration (BAC) of 0.08% more consistently than the NIAAA definition of consuming 5+/4 + drinks for males and females within this time interval (Lange and Voas, 2001, National Institute on Alcohol Abuse and Alcoholism, 2017, Read et al., 2008). Participants who reported ≥ 5 binge episodes within the previous 6 months with a binge episode in the past month were categorized as BDs (N = 19), while those who reported ≤ 2 binge episodes within that time window, and none within the past month, were identified as LDs (N = 18). Groups did not differ on age, gender, intelligence, and family history of alcoholism (see Table 1 for detailed group characteristics). Participants provided written informed consent and were reimbursed monetarily for their participation.

**Table 1:** Participant characteristics for binge and light drinking groups.

	**Binge (BD) (n = 19)**	**Light (LD) (n = 18)**	***U*/χ^2^**	**p-value**
Female %	57	50	.232^a^	.630
Ethnicity (white, non-Hisp) %	68	72	.201^a^	.366
Positive Fam. Hist. of Alcoholism %	63	56	.222^a^	.638
Age (yrs)	23.5 ± 3.1	25.6 ± 4.1	117	.105
WASI-II % rank (FSIQ-2)	68.2 ± 20.8	74.1 ± 21.4	132	.349
In the past six months Drinking days per week Drinks per occasion Drinks consumed per week Binge episodes Alcohol-related blackouts Max No. of drinks in 24 h	2.61 ± 1.14 5.42 ± 2.3 14.0 ± 7.7 14.3 ± 13.9 3.15 ± 2.7 12.5 ± 8.7	1.44 ± 1.01 2.50 ± 1.3 3.40 ± 2.7 0.40 ± 0.7 0.17 ± 1.0 3.4 ± 2.0	77 44 25 0 91 13	**.003** **<.001** **<.001** **< 0.001** **<.001** **<.001**
Age onset of alcohol use	16.2 ± 1.8	18.2 ± 2.0	71	**.006**
Alc. Use Disorder Ident. Test (AUDIT)	13.58 ± 5.9	3.94 ± 1.6	6	**<.001**
Alcohol cravings (PACS)	8.05 ± 4.8	2.89 ± 2.7	58	**<.001**
Consequences of alcohol consumption (B-YAACQ)	9.32 ± 6.4	2.61 ± 3.1	56	**<.001**
Alcoholism Severity (SMAST)	2.26 ± 2.3	0.72 ± 1.0	91	**.011**
Drinking motives (DMQ-R) Social Coping Conformity Enhancement	8.11 ± 1.13 5.06 ± 1.51 4.56 ± 1.72 6.89 ± 0.96	6.17 ± 1.29 3.61 ± 0.85 4.28 ± 1.32 5.17 ± 1.42	47 66 151 118	**<.001** **.002** .717 .155
Anxiety (GAD-7)	2.58 ± 2.6	2.89 ± 2.7	162	.775
Depression (PHQ-9)	3.32 ± 3.1	3.11 ± 2.9	166	.869
ADHD Symptomology (ASRS)	1.79 ± 1.7	1.17 ± 1.4	138	.288
Impulsivity (ABIS) Motor Attention Non-planning	8.94 ± 3.06 10.3 ± 2.40 8.00 ± 2.68	7.06 ± 1.80 9.22 ± 2.05 7.39 ± 2.03	104 118 145	.062 .155 .575
Sensation Seeking (BSSS) Experience Boredom Thrill Disinhibition	8.61 ± 1.5 4.11 ± 0.79 7.28 ± 1.46 3.83 ± 0.73	8.00 ± 1.68 3.61 ± 0.740 6.83 ± 2.48 3.00 ± 1.0	124 95 148 86	.208 **.029** .643 **.014**
Eysenck Personality (EPQ-R) Neuroticism Psychoticism Extraversion	3.72 ± 2.7 2.44 ± 2.0 9.56 ± 2.7	3.61 ± 3.7 2.28 ± 1.4 7.78 ± 3.5	95 161 114	.566 .961 .118

a Tested using chi-square; all other comparisons performed using non-parametric Mann-Whitney *U* test. Significant p-values reported with boldface font. WASI-II: Wechsler Abbreviated Scale of Intelligence; AUDIT: Alcohol Use Disorder Identification Test; PACS: Penn Alcohol Craving Scale; B-YAACQ: Brief Young Adult Alcohol Consequences Questionnaire; SMAST: Short Michigan Alcoholism Screening Test; DMQ-R: Drinking Motivations Questionnaire Revised; GAD-7: Generalized Anxiety Disorder; PHQ-9: Patient Health Questionnaire; ASRS: Adult ADHD Self-Report Scale; ABIS: Abbreviated Impulsiveness Scale; BSSS: Brief Sensation Seeking Scale; EPQ-R: Eysenck Personality Questionnaire Revised.

### 2.2 Experimental protocol

Participants completed a battery of questionnaires assessing details of frequency and quantity of alcohol consumption (Alcohol Use Disorder Identification Test, AUDIT, Saunders et al., 1993), the pattern of alcohol intake within the past thirty days (Time Line Follow Back, TLFB, Sobell and Sobell, 1996), intensity of craving (The Penn Alcohol Craving Scale, PACS, Flannery et al., 1999), behaviors associated with alcohol misuse (Short Michigan Alcohol Screening Test, SMAST, Selzer et al., 1975), motivations influencing drinking behaviors (Drinking Motive Questionnaire Revised Short Form, DMQ-R SF, Kuntsche and Kuntsche, 2009), and the frequency of detrimental outcomes as a consequence of drinking (Brief Young Adult Consequences Questionnaire, B-YAACQ, Kahler et al., 2005). Participants were also asked to rate the presence of depressive symptoms (Patient Health Questionnaire, PHQ-9, Kroenke and Spitzer, 2002), anxiety (Generalized Anxiety Disorder, GAD-7, Spitzer et al., 2006), attention deficit and/or hyperactivity characteristics (Adult ADHD Self-Report Scale, ASRS, Kessler et al., 2005), extent of impulsive attributes associated with motor, non-planning, and attentional characteristics (Abbreviated Brief Impulsivity Scale, ABIS, Coutlee et al., 2014), propensity for risk taking and/or sensation seeking behaviors (Brief Sensation Seeking Scale, BSSS, Hoyle et al., 2002), and affective personality traits (Eysenck Personality Questionnaire, EPQ, Eysenck and Eysenck, 1975). Cognitive abilities were measured with the Wechsler Abbreviated Scale of Intelligence (WASI-II, Wechsler, 1999). Family history of alcoholism was assessed using an abbreviated form of the Family History Assessment Module (FHAM, Rice et al., 1995). Positive family history for alcoholism (FH + ) was defined as having at least one immediate family member (father, siblings) and one immediate or extended relative (cousins, aunts, uncles) or 3 + extended family members with a prior diagnosis for AUD. Participants who reported maternal FH + were excluded to avoid possible fetal alcohol exposure confounds. On the day of scanning, participants were screened for the presence of illegal substances. Women were additionally screened for pregnancy and all tests were negative.

### 2.3 Experimental paradigm

Error-related behavioral and neural indices were examined using a modified variant of the Go/NoGo task (Garavan et al., 1999, Holcomb et al., 2019). Participants were presented with pseudorandomized sequences of X and Y letters and were asked to press a button using their right index finger (Go trials) for each stimulus alternation (e.g. X-Y-X-Y). NoGo trials required withholding of behavioral response to stimulus repetition (e.g. X-X or Y-Y) and were always followed by a Go trial, resuming behavioral response demands. Errors of commission or inhibitory control failures were defined as responses on NoGo trials. All stimuli were presented in white font on a black background for 230 ms every 1300 ms (±200 ms jittered in 50 ms increments). Participants completed 5 runs with a total of 900 trials comprising 750 (83%) Go and 150 (17%) NoGo trials. Participants were instructed to respond as quickly as possible while maintaining accuracy and to refrain from anticipatory button presses prior to stimulus presentation. Task administration was implemented via Presentation v.19.0 software (Neurobehavioral Systems) with transistor-transistor (TTL) pulses assuring synchronization of task onset with the beginning of each fMRI acquisition run. Stimulus sequencing was optimized using Optseq2 software (Dale, 1999) (https://surfer.nmr.mgh.harvard.edu/optseq/).

Structural and functional images were collected using a GE Discovery 750 3 T whole body scanner with an 8-channel head coil at the CFMRI Keck Center at University of California San Diego (UCSD). Structural T1-weighted images were acquired with a high-resolution spoiled gradient recalled echo (SPGR) imaging sequence using the following parameters: TR = 7.38 ms, TE = 2.984 ms, flip angle = 8°, FOV = 240 mm, matrix = 256 × 256, 170 axial slices with a 1.2 mm slice thickness, and an in-plane resolution of 0.94 × 0.94 mm. During task performance, functional blood oxygen-level dependent (BOLD) T2*-weighted images were collected with an echo planar imaging (EPI) sequence consisting of 35 interleaved axial oblique 4 mm thick slices covering the entire brain, that were aligned with anterior and posterior commissure (AC-PC) landmarks. Functional images were acquired using the following parameters: TR = 2000 ms, TE = 30 ms, flip angle = 90°, FOV = 220 mm, matrix 64 × 64, in-plane resolution of 3.44 × 3.44 mm.

### 2.4 Data analysis

#### 2.4.1 Behavioral measures and analysis

The high ratio of Go relative to NoGo trials in this task induced a strong response prepotency which increased the likelihood of inhibition failures on NoGo trials (Wessel, 2018b). BD and LD groups were compared on error rates and reaction times on errNoGo trials. To additionally examine post-error slowing (PES) effects, reaction times during Go trials immediately following corNoGo were compared to those after errNoGo trials within a mixed-design ANOVA with Group as the between-subject factor, and trial type as the within-subject factor. Cohen’s *d* was calculated as a standardized measure of between-group effect size (Cohen, 1988) using the G-power statistical analysis tool. Categorical variables were analyzed with the *χ^2^* test, whereas all other group differences were assessed with nonparametric Mann-Whitney *U* tests. All statistical analyses were carried out using SPSS 26 software.

#### 2.4.2 Analysis of event-related fMRI-BOLD signal to errors vs correct NoGo trials

Structural and functional images were examined using Analysis of Functional Neuro-Images (AFNI) software (Cox, 1996, Cox, 2012). Motion-related artifacts were mitigated by removing TRs where rotational and translational motion exceeded .3mm, and those in which ≥ 25% of voxels were identified as outliers. Structural and functional images were coregistered for each participant and then normalized to the MNI (TT_avg152TI) template provided by AFNI. A third-order polynomial accounted for signal drift and six motion parameters were used to regress out motion during deconvolution. A canonical hemodynamic response function (GAM) was used to model each trial. Spatial smoothing was performed with a three-dimensional Gaussian kernel (FWHM 8.0 mm) with voxels scaled to represent percent signal change prior to deconvolution. Go-related BOLD activation served as the baseline. Statistical maps were generated for each participant using 3dDeconvolve with a residual maximum likelihood (REML) and generalized least squares (GLSQ) analysis method to identify voxels with significant changes from baseline (Chen et al., 2012). Group-level analysis utilized a mixed-effects *meta*-analysis (MEMA) comparing coefficients and associated t-values generated by REML analysis. For between group differences, cluster simulations were carried out using the AFNI program 3dClustSim to determine the minimum cluster size at the whole brain level to control for family wise error. A voxel-wise p = .002, q = 0.05 (FDR) and cluster-wise p = .05, resulted in an estimated cluster size threshold of 29 contiguous voxels. Regions-of-interest (ROI) were identified from the significant voxel clusters based on the statistical map created from the BD > LD contrast for error-related activity (Friston et al., 2006, Poldrack, 2007). Beta coefficients representing percent signal change from baseline were extracted from each ROI for all participants. Nonparametric Spearman’s rank (rho) correlations were used to investigate associations between the error-related BOLD activity and variables related to task performance, alcohol intake, and personality/disposition. The Benjamini-Hochberg procedure was applied to correct for multiple correlations based on a false discovery rate approach (FDR = 0.05) (Hochberg and Benjamini, 1990).

#### 2.4.3 Event-related functional connectivity analysis

Functional connectivity analysis was carried out using CONN-fMRI toolbox v17 as implemented through SPM12. Pre-processing procedures followed CONN’s standard pipeline including slice-timing correction, realignment, co-registration, normalization to MNI space, and spatial smoothing steps (Whitfield-Gabrieli and Nieto-Castanon, 2012). A component-based noise correction procedure (Compcor) identified potential confounding temporal factors derived from estimates of subject-specific motion parameters, BOLD signal present in white matter and cerebrospinal fluid masks, and main condition effects (Go, errNoGo, and corrNoGo). Motion thresholds were set to CONNs default settings. Framewise displacements of 0.9 mm or greater, or global BOLD signal changes above 5 standard deviations, were flagged as outliers. The scrubbing/censoring procedure was performed with CONNs Artifact Detection Tool (ART), which flagged 1.2% of the data series as potential outliers across all participants. This was used as a regressor of no interest during the denoising step of analysis. Components representing temporal confounds were regressed from the BOLD time series at each voxel. The residual BOLD time series were then bandpass filtered at a frequency range of 0.008–0.09 Hz (Whitfield-Gabrieli and Nieto-Castanon, 2012). No significant correlations were found between functional connectivity measures and head motion.

Task conditions including corNoGo, errNoGo, and Go trials were modeled with subject-specific functional volumes and stimulus timing files used for initial BOLD analysis. Seed-to-voxel connectivity maps were generated for each participant. Bivariate correlations were used to determine the linear association of the BOLD time series between a seed and significant voxel clusters with a Fisher’s z transformation applied to the correlation coefficients. The same seeds were used across all participants and represented between-subject (q = 0.05) task-related BOLD contrasts based on errNoGo > corNoGo comparisons. This analysis made it possible to investigate group differences in cognitive control engagement during inhibition errors at the network level. ERFC was analyzed using a non-parametric permutation-based analysis approach across the entire brain volume (Bullmore et al., 1999, Whitfield-Gabrieli and Nieto-Castanon, 2012). ERFC was considered significant at joint-probability thresholds of 0.01 for height (peak voxel intensity) and 0.05 for cluster-extent with false-discovery rate correction for multiple comparisons. For significant voxel clusters, the REX toolbox was utilized to extract connectivity values (mean z-scores) for each participant (Whitfield-Gabrieli and Nieto-Castanon, 2012). These values were used to calculate Spearman’s rank (rho) correlations with variables related to task performance, alcohol intake, personality/disposition and were corrected for multiple correlations based on a false discovery rate using the Benjamini-Hochberg procedure (FDR = 0.05) (Hochberg and Benjamini, 1990).

## 3 Results

### 3.1 Participant characteristics

BD and LD groups did not differ on age, gender, ethnicity, FH+, or intelligence (Table 1). As anticipated, BDs reported consuming more alcohol, higher levels of craving, and more negative consequences of drinking. No group differences were observed on variables assessing anxiety, depression, ADHD, impulsivity, and personality traits, but BDs had higher scores on the measures of boredom and disinhibition.

### 3.2 Task performance

**Fig. 1:**
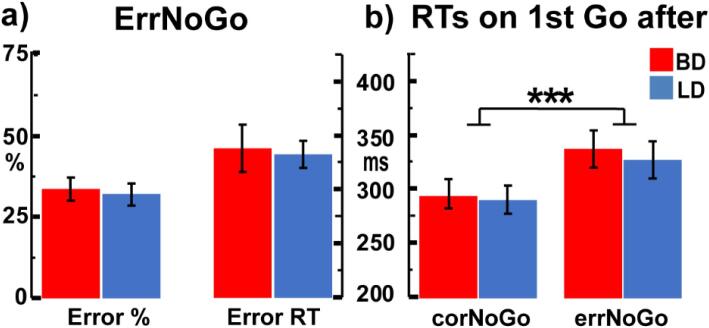
Task performance: a) NoGo error rates and errNoGo reaction times (mean ± standard errors). b) Reaction times for Go trials immediately following corNoGo and errNoGo trials. Both groups exhibited equivalent post-error slowing. *** p < .001.

BD and LD groups did not differ in the rate of inhibition failures on NoGo trials, *F_1,35_* = 0.113, p = .74, d = 0.11 nor in error-related reaction times, *F_1,35_* = 0.05, p = .83, d = 0.07 (Fig. 1a). As shown in Fig. 1b, post-error slowing (PES) was reflected in increased response latencies on Go trials immediately following errNoGo trials compared to those after corNoGo trials overall, *F_1,35_* = 36.75, p < .001, d = 0.62. Both groups demonstrated equivalent PES, *F_1,35_* = 0.104, p = .75, d = 0.02.

### 3.3 Error-related BOLD activity

**Fig. 2:**
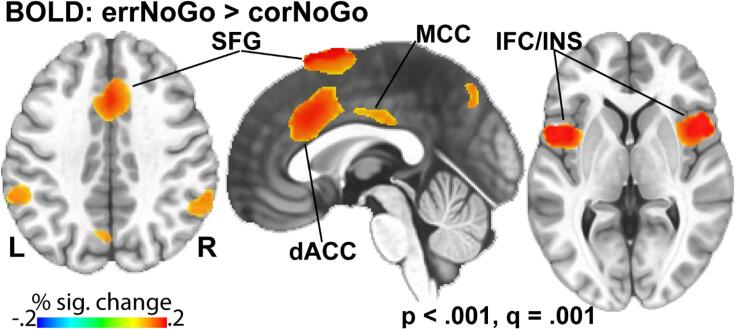
Across both groups, greater blood-oxygen level dependent (BOLD) activity was observed to errNoGo compared to corNoGo BOLD in distributed areas. BOLD contrast errNoGo > corNoGo, was thresholded at voxel-wise p < .001, with false discovery rate (FDR) correction q = 0.001, cluster-wise p = .05. ErrNoGo trials generated greater BOLD activation in the dorsal anterior cingulate (dACC), mid-cingulate (MCC), superior frontal gyrus (SFG), inferior frontal cortex (IFC) and insula bilaterally, and parietal cortices along with the precuneus, relative to corNoGo trials.

Across both groups, errNoGo trials generated stronger activation than corNoGo trials (Fig. 2), which was particularly notable in the inferolateral prefrontal and parietal cortices bilaterally, along with the dorsal anterior cingulate, mid-cingulate, supplementary motor area, and precuneus medially. This activation pattern aligns well with previously reported evidence on error-related activity (Dosenbach et al., 2006, Menon et al., 2001, Neta et al., 2015).

**Fig. 3:**
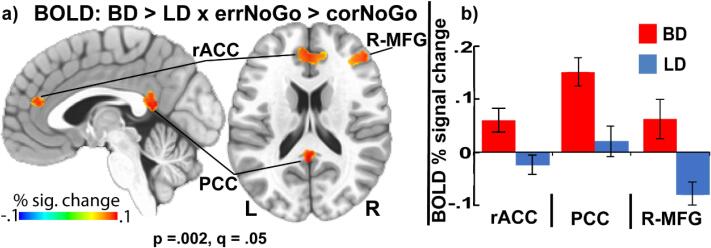
a) Group differences in BOLD activation for errNoGo vs.corNoGo contrast, thresholded at voxel-wise p = .002, with false discovery rate (FDR) correction q = 0.05, cluster-wise p = .05, with ≥ 29 contiguous voxels. BDs exhibited greater BOLD activation in the rostral anterior cingulate (rACC), posterior cingulate (PCC), and right middle frontal gyrus (R-MFG) compared to LDs. b) Mean ± SEM of BOLD % signal change for both groups and across these regions of interest (ROIs).

Group comparisons of errNoGo > corNoGo contrast revealed that BDs showed greater activation than LDs in the right middle frontal gyrus (R-MFG), *F_1,35_* = 10.64, p = .002, d = 1.1, rostral anterior cingulate (rACC), *F_1,35_* = 8.45, p = .006, d = 0.96 and posterior cingulate (PCC), *F_1,35_* = 11.33, p = .002, d = 1.1 (Fig. 3).

**Fig. 4:**
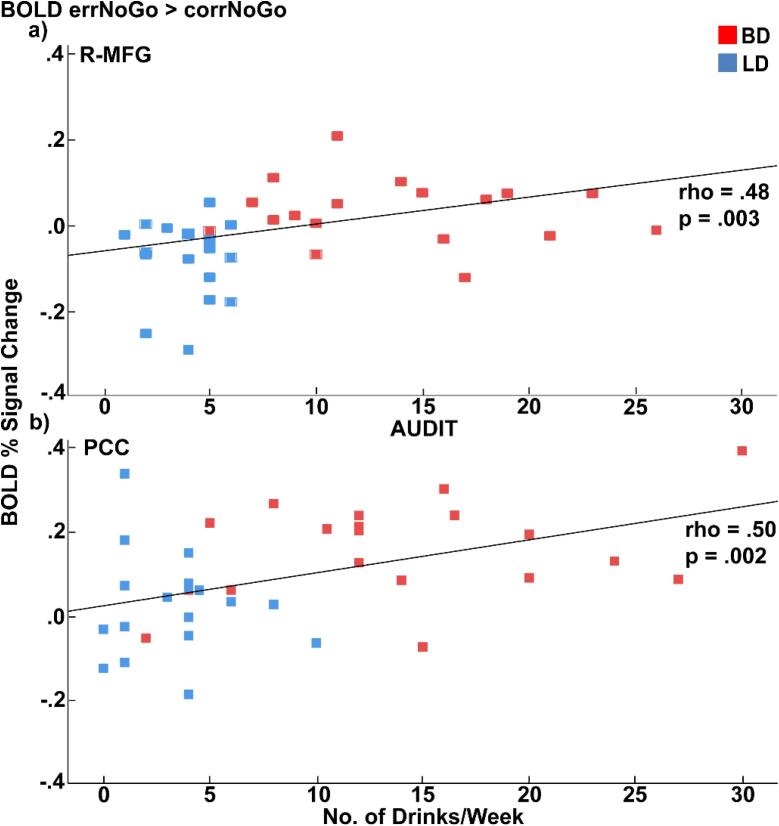
Scatter plots depicting correlations between errNoGo vs. corNoGo BOLD contrast and drinking indices as follows: a) R-MFG and AUDIT scores, b) PCC with average weekly alcohol intake for participants across both groups. R-MFG: right middle frontal gyrus; PCC: posterior cingulate cortex.

FDR-corrected Spearman’s rank correlations computed across all participants indicated that increased BOLD activity in the right middle frontal gyrus (R-MFG), rostral anterior cingulate (rACC), and posterior cingulate (PCC), was positively associated with a range of alcohol related variables including AUDIT scores, binge episodes, high-intensity drinking, weekly consumption, and dimensions of drinking motivations, range from rho = 0.47 to rho = 0.51, all p < 0.05. Representative examples of those correlations are shown in Fig. 4. Conversely, error-related BOLD activity was not associated with task performance nor with measures of personality or disposition.

### 3.4 Event-Related functional connectivity

**Fig. 5:**
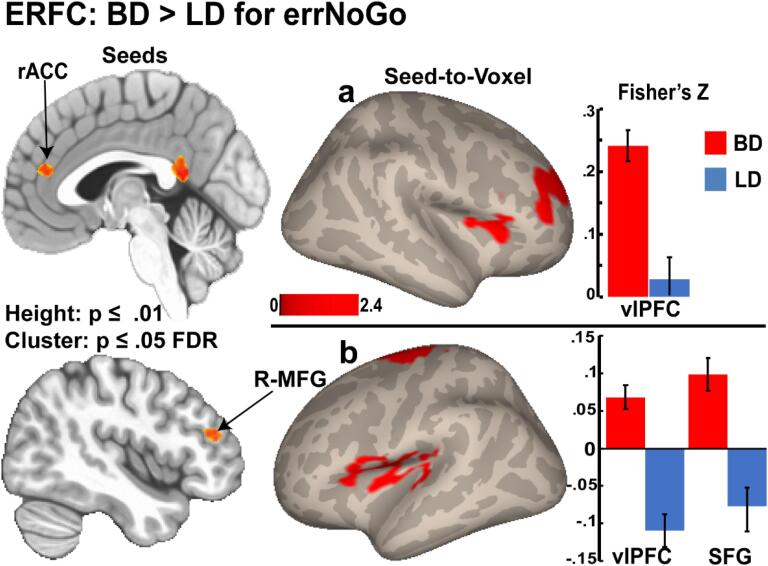
Seed-to-voxel connectivity maps displaying seed regions (left column), significant voxel clusters identified in connectivity analysis (middle column), and bar graphs of Fisher’s Z connectivity values for each cluster (right column). Relative to LDs, BDs show (a) increased connectivity between the rACC seed and voxel cluster in the right mid/inferior frontal cortex; (b) increased connectivity between the R-MFG seed and voxel clusters in left IFC and SFG. Connectivity indices are represented with a peak voxel threshold of p ≤ 0.01 for height and a cluster extent threshold of p ≤ 0.05, corrected for false-discovery rate (FDR), and carried out using a permutation based non-parametric approach.

BD and LD groups differed in their functional connectivity patterns during inhibition failures on errNoGo trials in all error-related seed ROIs used for seed-to-voxel analysis (Fig. 5, Table 2). Relative to LDs, BDs exhibited increased functional connectivity between the rostral ACC and the right lateral frontal cortex (Fig. 5a). BDs also showed greater connectivity between the R-MFG and the left ventrolateral cortex and the superior frontal cortex (SFG) (Fig. 5b).

**Table 2:** Seed-to-voxel analysis of functional connectivity during errNoGo in BD and LD groups.

Seed region	Cluster regions	Voxels in region	BD conn. Mean (SD)	LD conn. Mean (SD)	Cluster-size p-FDR corr.	Cohen’s *d*
rACC Cluster coord 34, 42, 32	R-FP R-FO R-Insula R-IFG R-Putam	869 74 47 59 46	0.24(0.11)	0.03(0.14)	0.001	1.65
R-MFG Cluster coord −46, 12, 08	L-Cent O L-Insula L-Putam L-HG L-FO L-Thalam L-Caudate L-Pallid	255 223 145 125 74 52 26 25	0.07(0.07)	−0.1(0.09)	0.003	2.16
R-MFG Cluster coord −18, −16, 68	L-Precg L-SFG L-SMA L-Postcg	414 413 201 9	0.1(0.09)	-0.08(0.12)	0.0309	1.58
						

Functional connectivity during errNoGo trials. First column: seed regions along with corresponding peak-voxel coordinates of clusters showing significant functional connectivity. Correction for false-discovery rate multiple comparisons was applied using a permutation based non-parametric approach across the entire brain volume with joint-probability thresholds = 0.01 for height and 0.05 for cluster-extent. Second column: the regions composing significant voxel clusters. The third column contains the number of voxels in each region comprising a cluster. Fourth and fifth columns list the mean connectivity values for each group across the entire cluster. Column six shows FDR-corrected cluster level p values. The rightmost column provides Cohens d effect sizes. FP: Frontal pole, FO: Frontal orbital, IFG: Inferior frontal gyrus, Putam: Putamen, Cent O: Central operculum, HG: Heschl's gyrus, Thalam: Thalamus, Precg: Precentral gyrus, SFG: Superior frontal gyrus, Postcg: Postcentral gyrus.

**Fig. 6:**
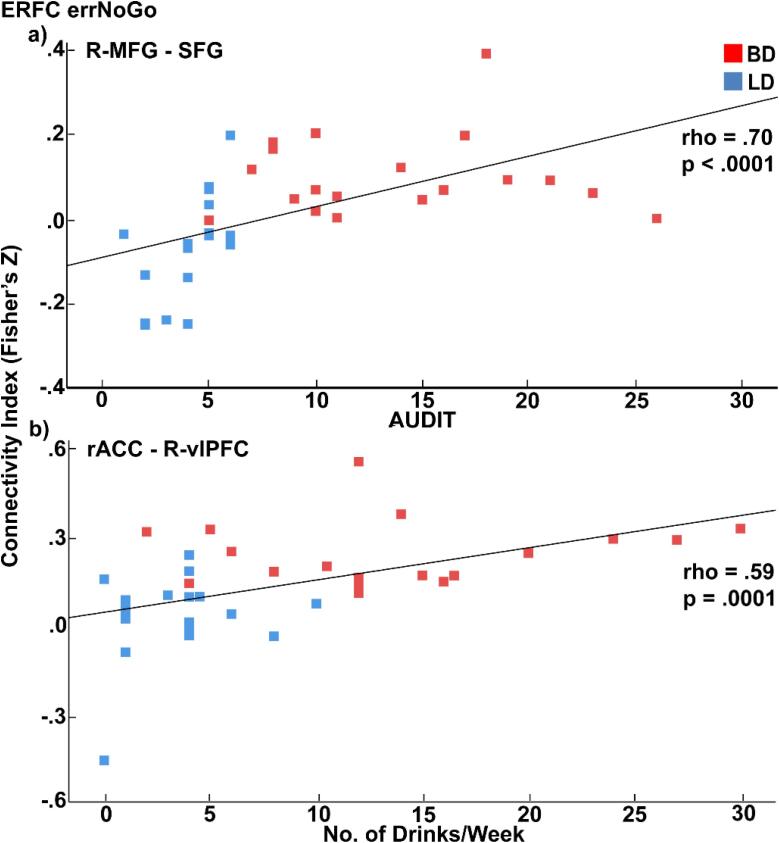
Scatter plots showing correlations between BOLD connectivity during errNoGo and drinking variables: a) right middle frontal gyrus (R-MFG) seed and superior frontal gyrus (SFG) with AUDIT scores; b) rostral anterior cingulate (rACC) seed and right ventrolateral prefrontal cortex (R-vlPFC) with average weekly alcohol intake.

Connectivity values for all seeds and across all participants revealed positive correlations with alcohol-related variables. The most robust correlations that survived FDR correction were again centered on the variables describing high intensity drinking behavior including AUDIT scores, average number of drinking days per week along with binge episodes, blackouts, and maximum number of drinks consumed in 24 h within the previous 6 months, and drinking motivations ranging from rho = 0.40 to rho = 0.72, all p < 0.01. Examples of these correlations are shown in Fig. 6. Variables related to task performance, personality, or disposition, did not correlate with functional connectivity values generated from any of the seeds used for analysis.

## 4 Discussion

The current study evaluated the task-related fMRI-BOLD activation and functional connectivity patterns during error processing in young adults engaging in binge drinking, compared to those who drink regularly but in low-risk patterns. The two groups showed comparable proficiency on a Go/NoGo task. As expected, the overall BOLD activity pattern to NoGo commission errors included the dACC and SFG medially along with the parietal cortices, vlPFC and adjoining insula bilaterally, confirming extant evidence. However, group comparisons on task-related BOLD activity and functional connectivity revealed several notable and novel findings which can be summarized as follows: 1) BDs demonstrated greater error-related BOLD response in the rACC and R-MFG relative to their LD counterparts; 2) BDs exhibited greater functional connectivity between these seeds and the frontolateral cortices than LDs; 3) The observed indices of task-related BOLD activity and ERFC were positively correlated only with alcohol intake, drinking motivations and habits, but not with measures of mood/disposition, cognitive capacity, or error-related behavioral measures. Taken together, these results are consistent with a compensatory interpretation as BDs show greater activity and engage an extended prefrontal network in the service of maintaining adequate task performance.

The two groups did not differ on task performance as reflected in equivalent NoGo error rates and reaction times (Fig. 1a). Even though PES was confirmed on Go trials following errors (Danielmeier and Ullsperger, 2011), it characterized both groups equally (Fig. 1b), which is consistent with a similar observation on a Go/NoGo task in individuals with AUD (Li et al., 2009). This suggests that PES, as a form of proactive, anticipatory control, seems to be robust to binge or heavy drinking patterns, although diminished PES has been reported for other types of addiction, such as cocaine use disorder (Sullivan et al., 2019). The lack of BD vs LD differences on behavioral measures is in line with a growing body of evidence indicating that they may be less sensitive than neural indices that typically detect group differences (Campanella et al., 2017, Cohen-Gilbert et al., 2017, Correas et al., 2019, Crego et al., 2012, Crego et al., 2009, Holcomb et al., 2019, Huang et al., 2018, Lannoy et al., 2019, López-Caneda et al., 2012, López-Caneda et al., 2017, Petit et al., 2013, Schweinsburg et al., 2010), and are predictive of increased alcohol and substance use over time (Courtney et al., 2020, Moeller et al., 2016)

The overall BOLD activation pattern to NoGo errors (Fig. 2) is consistent with extensive evidence demonstrating that a distributed, predominantly frontal network subserves error processing as a key dimension of cognitive control (Dosenbach et al., 2006, Krönke et al., 2018, Manoach and Agam, 2013, Neta et al., 2015, Ullsperger et al., 2014, Wessel, 2018a). Errors are essential for establishing adaptive loops and for allowing adjustments in the service of goal-relevant optimization (Hoffmann and Beste, 2015, Wessel, 2018a). The observed dACC activity confirms its role as a central node in performance monitoring and error processing (Botvinick et al., 2004, Carter and van Veen, 2007, Dehaene et al., 1994, Garavan et al., 2002, Heilbronner and Hayden, 2016, Kolling et al., 2016, Marinkovic et al., 2012, Mathalon et al., 2003, Neta et al., 2015, Ridderinkhof et al., 2004b). Complementary evidence is provided by EEG studies that estimate the generators of error-related negativity (ERN) to the dACC (Trujillo and Allen, 2007). The vlPFC is also consistently engaged by tasks probing inhibitory control and attentional capture (Aron et al., 2014, Chikazoe, 2010, Correas et al., 2019, Forstmann et al., 2008, Hampshire, 2015). The tight coupling between the medial and ventrolateral PFC and other areas may orchestrate goal-oriented behavior in the context of performance monitoring (Botvinick et al., 2004, Kerns et al., 2004, Ridderinkhof et al., 2004a).

### 4.1 Error-related neural dysregulation in BDs: Comparisons with AUD samples

In the present study, the BD group showed greater BOLD activity to error NoGo trials in the rACC, PCC, and the right dorsolateral PFC, compared to the LD group (Fig. 3). Studies exploring error-related activity in BDs are lacking. A lone report used a Go/NoGo variant with letters superimposed on alcohol-related vs. neutral background images (Campanella et al., 2017). Across all types of cues, the study reported diminished error-related BOLD activation of the ACC and right lateralized inferior frontal cortex but heightened activity of the amygdala and occipital cortices in BDs relative to LDs (Campanella et al., 2017). However, considerable differences in multiple aspects of the two respective paradigms preclude direct comparisons with the current study. In contrast, the present results appear to closely agree with a study investigating individuals diagnosed with AUD. Using a Stop-signal task, Li et al. (2009) reported greater BOLD activation in response to Stop-errors in regions analogous to the rACC and R-MFG observed in the present study. These parallel findings suggest that the enhanced BOLD activity to failures of inhibitory control observed in both BD and AUD groups may play a compensatory role serving to maintain adequate task performance on tasks probing cognitive control (Chanraud et al., 2013, Molnar et al., 2018).

The concept of compensatory neural engagement is rooted in the theory of neural reserve or the ability to perform adequately despite neural damage as it occurs in aging (Bartrés-Faz and Arenaza-Urquijo, 2011, Barulli and Stern, 2013). In this view, greater activation reflects the compensatory engagement of neural circuits to maintain normative performance with functional deficits becoming apparent as brain reserve is diminished (Staff, 2012). Increased prefrontal activation has been reported in studies of people with AUD during tasks probing response interference (Wilcox et al., 2015), working memory (Caldwell et al., 2005, Desmond et al., 2003, Pfefferbaum et al., 2001), and response inhibition (Czapla et al., 2017, Karch et al., 2008), consistent with our results. A series of studies conducted by Chanraud and colleagues reported compensatory activation in participants with AUD who performed cognitively demanding executive tasks (Chanraud et al., 2013, Chanraud et al., 2011, Chanraud et al., 2010, Chanraud and Sullivan, 2014). Furthermore, compensatory network reorganization with increased prefrontal activation during face processing has been observed in participants with AUD (Marinkovic et al., 2009, Oscar-Berman and Marinković, 2007). Even though neuroimaging studies in binge drinkers are scarce, the extant evidence suggests that adequate behavioral performance may be maintained by increased engagement of prefrontal regions in a compensatory manner, particularly during tasks imposing greater cognitive demands (Ames et al., 2014, Kashfi et al., 2017, Molnar et al., 2018, Squeglia et al., 2012, Squeglia et al., 2011, Wetherill et al., 2013).

### 4.2 Inhibition errors engage the rostral anterior and posterior cingulate cortices in BDs

As shown in Fig. 3, the rACC area showed greater activity specifically to inhibition failures (errNoGo vs corNoGo contrast) in BDs compared to LDs, which is suggestive of error-induced affective engagement of limbic circuitry. A growing body of evidence implicates the rACC in processing affective components of error-related activity (Bush et al., 2000, Kragel et al., 2018, Luu et al., 2003, Taylor et al., 2006). Indeed, errors are accompanied with negative affect, which plays a role in behavioral adjustments and emotion regulation (Dignath et al., 2020, Wessel, 2018a). Errors occur unexpectedly and have aversive quality, which is known to elicit an “oh, no!” orienting response reflected in increased autonomic arousal (Hajcak et al., 2003, Wessel, 2018a). When autonomic responses are measured simultaneously with fMRI-BOLD during the Stroop task, greater rACC activity is elicited by errors accompanied with pupil dilation, which is indicative of sympathetic arousal (Critchley et al., 2005). The rACC is also associated with emotional regulation of negative affect that accompanies errors (Ichikawa et al., 2011). Relatedly, the peak ACC activation on inhibition error trials accompanied with higher levels of self-reported frustration, is located rostrally to the canonical dACC activation to errors (Spunt et al., 2012). Even though the emotion and cognition are highly integrated (Pessoa, 2018), neuroimaging evidence supports the functional dissociation between the rostral and dorsal ACC during error processing or tasks probing affective functions (Bush et al., 2000, Mohanty et al., 2007, Steele and Lawrie, 2004). In the present study, the observed peak location of the error-specific activity group differences (errNoGo vs corNoGo contrast), is aligned with such evidence and is indicative of higher responsivity to errors in BDs. Within this framework, the dACC monitors for error-related conflict in concert with the lateral frontal cortex, whereas the rACC may contribute to emotional evaluation via integrated engagement of limbic structures (Inzlicht et al., 2015, Ullsperger et al., 2014, Wessel, 2018a). This area is well positioned to subserve error-related activity due to its rich interconnectivity with distributed subcortical and cortical structures (Etkin et al., 2006, Kunishio and Haber, 1994, Polli et al., 2005, Polli et al., 2009, Van Hoesen et al., 1993). Overall, increasing dysregulation of this region over time may play a role in development of the dampened emotional sensitivity that has been previously observed in BD and AUD samples (Huang et al., 2018, Marinkovic et al., 2009).

Our finding of greater PCC activation to errors in BDs is comparable to those of Schulte et al. (2012). They reported greater PCC activity in individuals with AUD during a demanding response switching condition in a modified version of the Stroop task, which is likely to induce errors. The elevated PCC activity was associated with alcohol-related variables. In the present study, the PCC activity was also positively correlated with drinking severity and alcohol intake, suggesting that dysregulation in this region can occur even after a relatively short span of heavy alcohol use. Though there is appreciable uncertainty regarding the functional role of the PCC, neuroimaging evidence confirms its contributions to error processing. Indeed, greater activity of the PCC has been observed on error trials (Wittfoth et al., 2008), as well as on trials immediately preceding inhibition errors (Li et al., 2007). A multimodal imaging study has implicated the PCC in the cognitive control circuitry of error monitoring by (Agam et al., 2011), confirming that error processing is subserved by both, the PCC and dACC.

### 4.3 Error processing in BDs is dependent on flexible and compensatory network engagement

Emphasizing the importance of efficient communication, recent models propose that cognitive control relies on flexible engagement of brain regions into cohesive networks to adaptively respond to contextual demands, with different areas of the PFC playing essential and complementary roles in that process (Braun et al., 2015, Bullmore and Sporns, 2009, Bullmore and Sporns, 2012, Miller and Cohen, 2001, Spielberg et al., 2015). Errors are rare, unpredictable, and aversive, and are processed by distributed, predominantly prefrontal cortical areas that subserve not only error detection, but also facilitate post-error modification (Danielmeier and Ullsperger, 2011, Dosenbach et al., 2006, Marinkovic et al., 2013, Neta et al., 2015). The current study found that inhibition errors elicited greater connectivity between the medial and lateral, primarily frontal areas in BDs compared to LDs, which correlated exclusively with alcohol-related variables (Fig. 5, Fig. 6, Table 2). Specifically, the BD group showed greater functional connectivity between the rACC and right lateral frontal cortex, as well as the R-MFG with the left ventrolateral cortex and the SFG. These findings provide additional support for the synchronous interaction between the medial and bilateral frontal cortices in the service of flexible and coordinated cognitive control of behavior (Botvinick et al., 2004, Dosenbach et al., 2007, Kerns et al., 2004, Marinkovic et al., 2019, Smith et al., 2019). Furthermore, they are consistent with previous reports of compensatory increase of neural activity in BDs during tasks probing cognitive control (Kashfi et al., 2017, Molnar et al., 2018), or working memory (Squeglia et al., 2011). Currently, there appears to be no studies that have investigated network connectivity in young adult BDs during cognitive tasks, and none focusing on error processing. Similarly, none of the ERFC studies in people with AUD have specifically investigated inhibition errors, although they have provided evidence on dysregulation of functional networks associated with cognitive control (Chanraud et al., 2011, Schulte et al., 2012).

Considering the importance of better insight into dysregulation of cognitive control networks in BDs, it is unfortunate that connectivity studies are exceedingly rare and focus exclusively on resting state activity (Arienzo et al., 2020, Herman et al., 2019, Sousa et al., 2019). Previously published examination of RSFC in the current sample of BDs (Arienzo et al., 2020), revealed enhanced connectivity between striatal reward regions with the orbitofrontal cortex and the rACC. Conversely, reduced connectivity between the inferior frontal cortex and hippocampus was indicative of disruption to networks associated with top-down control of behavior. Both findings were associated with a range of alcohol-related variables (Arienzo et al., 2020). Executive networks have demonstrated particular sensitivity to periods of high intensity alcohol use as greater connectivity was observed in a BD sample in the left executive control network while at rest (Sousa et al., 2019). In contrast, reduced connectivity within the ventral attention network, has been observed in higher drinking BDs (Herman et al., 2019). Taken together, these results provide evidence of the susceptibility of the PFC to neuroadaptation in association with binge drinking during early adulthood. More recently, evidence from animal research has indicated widespread reorganization of neurocircuitry, reflected in greater neural coactivation in alcohol-dependent and non-dependent mice compared to naïve animals (Kimbrough et al., 2020). This suggests that even moderate drinking may incite a pattern of brain activity that over time reinforces alcohol seeking behaviors and increases the likelihood of alcohol dependence (Kimbrough et al., 2020).

### 4.4 Allostatic changes may contribute to compensatory increase in BOLD activity and ERFC in BDs

In the absence of group differences in task performance, BDs showed greater BOLD activity during error processing in the present study. This finding is consistent with similar reports of increased activity in during tasks probing cognitive control in BD (Kashfi et al., 2017, Molnar et al., 2018, Wetherill et al., 2013) and AUD groups (Chanraud et al., 2013, Schulte et al., 2012). Furthermore, BDs exhibit greater connectivity between the medial and lateral prefrontal areas activated during error processing. Studies on AUD are broadly consistent with this finding, as AUD groups show greater connectivity with the regions outside of the typical activity pattern displayed by control groups (Chanraud et al., 2011, Schulte et al., 2012). Greater BOLD activity and functional connectivity observed in BD and AUD groups during cognitively taxing tasks are commonly interpreted as compensatory engagement to maintain normative performance (Chanraud et al., 2011, Molnar et al., 2018, Schulte et al., 2012). Even though the findings indicative of associations between increased neural activity and behavioral measures are limited, the available evidence supports the compensation hypothesis. In the present study, NoGo accuracy was positively correlated with mPFC BOLD activation (r_s_ = 0.45, p = .05) in the BD, but not LD group (r_s_ = -0.049, p = .85). Similarly, Chanraud et al. (2013) reported a positive relationship between accuracy on a working memory task and BOLD activity in the right middle frontal cortex in AUD participants (r = 0.71, p = .003). In a study on binge drinking, Molnar and colleagues (2018) reported a positive correlation between the Stroop task difficulty and the right ventrolateral prefrontal cortex activity to cognitive conflict (r_s_ = 0.47, p = 0.013). Taken together, this evidence is aligned with the compensatory account whereby activation increase and expanded network recruitment serve the purpose of compensatory engagement to offset the underlying deficits in association with alcohol misuse (Chanraud et al., 2013, Chanraud and Sullivan, 2014).

These findings are also consistent with the idea that functional dysregulation spurred by different forms of alcohol misuse reflects basic-level neuroadaptation. A mechanistic model of allostasis (Koob and Le Moal, 2008) outlines the neural underpinnings of compensatory change at the level of cell signaling and provides a supplementary interpretational framework. It is well established that acute alcohol intoxication increases GABA-mediated inhibition and reduces glutamatergic excitation, tipping the excitatory/inhibitory (E/I) neural balance towards inhibition (Kumar et al., 2009, Most et al., 2014, Roberto and Varodayan, 2017). Habitual drinking at hazardous levels elicits countervailing changes reflected in downregulated inhibitory and upregulated excitatory cell signaling. As a result, the E/I neural balance is tipped towards neural hyperexcitability in sober state, which persists beyond a binge episode (Correas et al., 2021, Most et al., 2014, Roberto and Varodayan, 2017). Studies using magnetic resonance spectroscopy (MRS) in conjunction with fMRI-BOLD are of particular relevance, because they show that GABA concentration is negatively associated with task-related BOLD activity (Donahue et al., 2010, Duncan et al., 2014, Hu et al., 2013), as well as ERFC (Chen et al., 2019, Sampaio-Baptista et al., 2015). This means that neural hyperexcitability is reflected in greater BOLD activity. Indeed, lower levels of GABA have been reported in young adult BDs in the ACC, in association with worse response inhibition and more negative consequences of alcohol use (Silveri et al., 2014). Using multimodal imaging methods that are directly sensitive to synaptic currents, we have recently reported that sober BDs show greater neural excitability than LDs, which correlated with their drinking levels (Correas et al., 2021, Most et al., 2014, Roberto and Varodayan, 2017). This is indicative of E/I imbalance and neural hyperexcitability in BDs, and provides supportive evidence for the mechanistic model of allostasis (Koob and Le Moal, 2008). Even though more research is needed to integrate the findings of altered neurotransmission with the changes at the level of functional networks in BDs, the allostatic model provides a principled physiological framework consistent with the greater BOLD activation and functional connectivity indices observed in the current study. Furthermore, the associations between lower GABA levels and regional and network-level fMRI-BOLD activity (Donahue et al., 2010, Duncan et al., 2014, Gao et al., 2015, Hu et al., 2013) aligns with potential applicability of the allostatic model to compensatory engagement of extended networks resulting from excessive alcohol consumption (Chanraud et al., 2013, Chanraud and Sullivan, 2014, Marinkovic et al., 2009, Molnar et al., 2018, Spielberg et al., 2015).

## 5 Conclusions

Using the spatial accuracy afforded by fMRI methods, we identified enhanced BOLD activation of the rACC, R-MFG, and PCC to inhibition failures in BDs when compared to LDs. When used as seeds, the rACC and R-MFG additionally showed greater connectivity with distributed prefrontal areas in BDs during error processing. Indices of error-specific BOLD activity and functional connectivity were positively correlated with a range of variables related to alcohol consumption, but not with those related to mood, disposition, task performance, or cognitive capacity. This evidence suggests that cognitive control, as reflected in performance monitoring, may be impacted by binge drinking in young adults. The lack of behavioral deficits in task performance suggests that expanded network engagement may have served a compensatory role to maintain efficiency of inhibitory control. The compensatory activity increase is consistent with hyperexcitability of neurotransmission proposed by the mechanistic model of allostasis (Koob and Le Moal, 2008) and confirmed in BDs (Correas et al., 2021, Most et al., 2014, Roberto and Varodayan, 2017). It needs to be noted that the question of whether the observed effects reflect premorbid traits or are a result of heavy drinking cannot be addressed by the current study which is cross-sectional in nature. A prospective, longitudinal approach would be needed to investigate such possibilities (https://abcdstudy.org/). However, all BOLD-related and ERFC indices correlated only with alcohol-related variables, suggesting that protracted alcohol misuse may be associated with alterations in neural function, particularly since the individuals most likely to engage in binge drinking are young and vulnerable to neurotoxicity (Crews et al., 2016, Cservenka and Brumback, 2017, Jacobus and Tapert, 2013, Petit et al., 2014b, Squeglia et al., 2014, Stephens and Duka, 2008). These findings are aligned with prominent models of addiction that have accentuated importance of executive functions in maintaining low-risk drinking levels via top-down control over behavior (Goldstein and Volkow, 2011, Koob and Volkow, 2016). The observed dysregulation of regions and networks associated with executive function in BDs provide evidence of potential indicators for those at risk of advancing toward AUD and may prove useful for developing clinical interventions designed to identify and mitigate alcohol misuse.

### CRediT authorship contribution statement

**Austin B. Alderson Myers:** Conceptualization, Data curation, Formal analysis, Investigation, Methodology, Visualization, Writing – original draft, Writing – review & editing. **Donatello Arienzo:** Conceptualization, Formal analysis, Methodology, Validation. **Sean M. Molnar:** Conceptualization, Data curation, Methodology, Validation. **Ksenija Marinkovic:** Conceptualization, Data curation, Funding acquisition, Investigation, Methodology, Writing – original draft, Writing – review & editing, Validation.

## Declaration of Competing Interest

The authors declare that they have no known competing financial interests or personal relationships that could have appeared to influence the work reported in this paper.
